# Innovative health service delivery models in low and middle income countries - what can we learn from the private sector?

**DOI:** 10.1186/1478-4505-8-24

**Published:** 2010-07-15

**Authors:** Onil Bhattacharyya, Sara Khor, Anita McGahan, David Dunne, Abdallah S Daar, Peter A Singer

**Affiliations:** 1 Li Ka Shing Knowledge Institute, 30 Bond Street, First Floor, Toronto, ON M5B 1W8, Canada; 2 Rotman School of Management, University of Toronto, 105 St. George Street, Toronto, ON, M5 S 3E6, Canada; 3 MaRS Centre, 101 College Street, 4th Fl/South Elevators, Suite 406, Toronto, ON M5G 1L7, Canada; 4 McLaughlin- Rotman Centre for Global Health, MaRS Centre, South Tower, 101 College Street, Suite 406, Toronto, ON, M5G 1L7, Canada

## Abstract

**Background:**

The poor in low and middle income countries have limited access to health services due to limited purchasing power, residence in underserved areas, and inadequate health literacy. This produces significant gaps in health care delivery among a population that has a disproportionately large burden of disease. They frequently use the private health sector, due to perceived or actual gaps in public services. A subset of private health organizations, some called social enterprises, have developed novel approaches to increase the availability, affordability and quality of health care services to the poor through innovative health service delivery models. This study aims to characterize these models and identify areas of innovation that have led to effective provision of care for the poor.

**Methods:**

An environmental scan of peer-reviewed and grey literature was conducted to select exemplars of innovation. A case series of organizations was then purposively sampled to maximize variation. These cases were examined using content analysis and constant comparison to characterize their strategies, focusing on business processes.

**Results:**

After an initial sample of 46 studies, 10 case studies of exemplars were developed spanning different geography, disease areas and health service delivery models. These ten organizations had innovations in their marketing, financing, and operating strategies. These included approaches such a social marketing, cross-subsidy, high-volume, low cost models, and process reengineering. They tended to have a narrow clinical focus, which facilitates standardizing processes of care, and experimentation with novel delivery models. Despite being well-known, information on the social impact of these organizations was variable, with more data on availability and affordability and less on quality of care.

**Conclusions:**

These private sector organizations demonstrate a range of innovations in health service delivery that have the potential to better serve the poor's health needs and be replicated. There is a growing interest in investing in social enterprises, like the ones profiled here. However, more rigorous evaluations are needed to investigate the impact and quality of the health services provided and determine the effectiveness of particular strategies.

## Introduction

There is a need for improved health services for the 2.6 billion people living on less than $2 a day [1]. The poor experience considerable barriers to health care such as limited purchasing power and health insurance, low health literacy, and residence in slums or remote rural areas which are frequently underserved [2]. These barriers must be considered in the way services are marketed, financed and delivered to this group to ensure that quality care is made available and affordable to the poor.

In part due to gaps in public health services, the private provision of health care has grown [3]. The presence of private health providers in low- and middle-income countries (LMIC) is significant. Recent estimates suggest that poor people seek care in the private sector for 35-95% of cases of childhood diarrheal and respiratory illnesses across a wide range of countries [4]. Private provision of care is not without its critics. The main concerns about private health care delivery are the underprovision of public goods in free markets, lack of access to care for the indigent, and the potential for providers to induce demand for unnecessary services to generate profit [5]. However, since public health services are not always available or in some cases perceived to be of poor quality, private health care delivery has been widely used in LMIC. It is therefore worthwhile to understand the private sector's potential contribution to health systems.

One area where the private sector may contribute is as a source of "disruptive innovators" - organizations who develop simpler and cheaper services that enable the participation of new sets of consumers previously excluded from conventional markets[6]. Providers in the private sector may operate on a for-profit or a not-for-profit basis[7], but there is a growing number of social enterprises which aim to develop models of pattern-breaking social change that can scale up easily, which can include novel financial strategies [8]. These social entrepreneurs attempt to improve the affordability, availability or quality of care for the poor. While this topic is of growing interest, the range of existing strategies used by the private sector has not been fully described. Recent reviews have either focused on specific strategies to engage the private sector (concluding, on the whole, that there is no rigorous evidence of benefit) or described a few organizations, focusing on commercial viability and not analysing a series of cases highlighting the range of mechanisms for improving care for the poor, as we do here[9-11].

Our goal is to describe a series of high profile social enterprises, describe the areas of innovations in their health service delivery models, and explore the potential of these models to create more inclusive and effective health services in resource-limited settings.

## Methods

### Selection of case studies

We searched MEDLINE for peer-reviewed articles, searched the grey literature including web sites, and contacted experts on the health systems of LMIC to identify private sector organizations considered to be examplars of business model innovation in health service delivery for the poor. We adopted Weberg et al.'s definition of innovation in health care, which emphasizes on the impact of the innovation on the market or population: "Innovation is something new, or perceived new by the population experiencing the innovation, that has the potential to drive change and redefine healthcare's economic and/or social potential" [12]. The "newness" in an innovation can be achieved by "recombining old ideas in a new way, creating a new process or product, using a process from another industry in one that has not used that process, or reordering an organization in a new and different way" [12]. Business models consist of four components: i) a product or service; ii) managers that bring together a set of resources required to deliver the product or service; iii) processes where employees and resources work together to repeatedly generate the product or service; and iv) a profit formula to ensure that the costs of the resources and processes are covered[6]. Health service delivery models are business models adopted in the provision of health services. We focused on organizations that employed innovative health service delivery models to bring about positive social impact, i.e. to improve affordability, accessibility and/or quality of health services for the poor, particularly those that had expanded beyond pilots, and had detailed descriptions of their strategies.

From an initial sample of 46, only six had sufficient information on their activities and impact in the peer-reviewed and grey literature for initial inclusion in this study (See Additional file 1 for search strategy). We attempted to contact the other 40 organizations, and we received ten replies. Structured, open-ended surveys were sent to these ten organizations and staff members were interviewed where possible to complement available information[7]. After reviewing the compiled information on their business models, we used purposive selection to eliminate organizations with very similar business strategies from the same geographical regions and/or disease areas in order to maximize variation and to highlight a wide range of activities. We further excluded those who did not provide health services directly. This left 10 organizations, 6 from the original search and 4 from the surveys. Table 1 lists the selected organizations and describes their scope of services, social impact and sources of funding. Case studies for each of these organizations were developed based on a content analysis of information from sources such as peer-reviewed literature, technical reports, external evaluations, web sites, news articles and interview results. The quality of the data for each organization was variable, as shown in Table 1. Most of the evidence comes from self-reported reviews on websites and published reports. Only half of the organizations have third-party evaluations on their performance and often social impact was inferred from the available information. For example, information on availability was based on descriptions of the volume and reach of services (e.g. within poor areas), data on affordability was based on pricing strategies, while information on quality (either technical quality or patient experience) was based on comparisons with existing services or use of strategies for quality improvement such as training, monitoring and evaluating.

**Table 1:** Innovative Private Sector Organizations Benefiting the Poor

Organization (Country/Year Started) *Scope of services*	Overall performance	Social Impact		↑ Improved ↔ No change ? Unknown	Quality of Evidence	Sources of Funding
				
		Availability	Affordability	Quality of Care		
**Aravind Eye Care System** (India/1976) *Eye Care Services *Manufacture of intraocular lenses; cataract surgery; vision screening	Largest and most productive eye care facility in the world; 2.5 million have received outpatient eye care and > 300,000 have undergone eye surgeries from April 2009 to March 2010	↑ Increased availability of services to rural areas through outreach camps, internet kiosks and vision centers	↑ Cost of cataract surgery reduced to $25; 70% of patients receive care subsidized or free	↑ High quality of services, with lower infection rate than UK	Self-reported evaluations; externally reviewed publications	Local entrepreneur

**Dentista Do Bem** (Brazil/2002) *Dental Care for youths: *Free treatment provided by existing practitioners	Reached > 12,000 children in 27 states in Brazil in 2009; model is being replicated in 6 Latin American Countries	↔ Existing practitioners provide free services	↑ Services provided by existing providers for free to poor youth	? Use of existing providers; provide systematic follow-up and feedback to ensure quality of care and motivate dentists	Self-reported questionnaire and review; foundation website	Local entrepreneur supported by partnerships with dentists and fundraising

**Greenstar Social Marketing Pakistan** (Pakistan/1991) *Reproductive and child health: *Education; intervention, monitoring and evaluation	2^nd ^largest family planning provider after the Government in Pakistan with a franchise network of over 7,500 active providers	↑ Outreach workers reach over 2.5 million people every year	↑ Serves higher proportion of poor clients than the government and provide over 26% of all modern contraceptives at affordable prices	↑ Continuous training and monitoring result in higher quality services than existing private facilities	Self-reported review and questionnaire; third party evaluation	Initially funded by international NGO with support from various government and private foundations and user fees

**Jaipur Foot** (India/1968) *Lower limb prosthetic: *manufacture and fitting	Distributed > 200,000 artificial limbs in India and > 13,000 in 18 other countries	↑ Distribution through clinics and outreach camps, 24 hours a day	↑ Reduced cost of a prosthetic leg and fitting to $35; prosthetics are distributed to clients for free	↑ Prosthetics are designed to meet the daily needs of the poor; focuses on customer orientation and quality service delivery	Self-reported statistics; third party evaluation	Local entrepreneur supported by local government and donations

**K-MET** (Kenya/1995) *Maternal and child care: *Trains existing providers on reproductive health, family planning, safe abortion care	Network of 204 health providers and community-based workers	↑ Provides care for rural communities where government services are unavailable	↑ Serves clients slightly poorer than community average; services benefit all income quintiles	↑ Gives loans to clinics and provides training to improve facilities and ensure safety and high quality of care	Externally reviewed publications; third party evaluation	Local NGO with support from donations and international grants

**Narayana Hrudayalaya Heart Hospital (NH)** (India/2001) *Coronary artery disease: *Heart surgeries and cardiac care	The 800-bed hospital performs high quality surgeries with eight times more volume than average Indian hospitals	↑ High volume hospital; 54 telemedicine centers, outreach camps and buses reach out to the rural poor	↑ High-volume strategy allowed NH to reduce cost of cardiac surgery to Rs 65,000 from Rs 150,000 (average Indian private hospital); 18% of patients receive care subsidized and 1% free	↑ Ensures high quality and efficient services by training surgeons and nurses, use of top-quality equipment; higher overall success rate in coronary artery bypass surgery than the U.S average	Self-reported review; externally-reviewed publications; third-party evaluations	Local entrepreneur with the help of capital funding from family members and Asia Heart Foundation plus user fees

**Population and Community Development Association (PDA)** (Thailand/1974) *Family planning and HIV/AIDS care*: Education; contraceptive/vasectomy/pregnancy termination services	Contributed to the decrease of Thailand's population growth rate from 3.3% in 1970 s to 0.6% in 2005; helped establish national HIV/AIDS prevention program in Thailand which reduced potential new infections by 90%; model adopted by the governments of many countries	↑ Nation-wide public education campaigns; outreach and mobile clinics reach 10 million Thais in 18,000 villages and poor urban communities; provide blood tests, family planning and pregnancy termination services for the poor where services were previously unavailable	↑ Most services are free; owns innovative commercial ventures to fund community health and development projects	? Quality of care unclear; aims to improve safety of services(e.g. reinforced safe abortion practices etc) and provides health education to the public	Self-reported review; Gates Awards press release; published reports	Local entrepreneur with support through donations and revenue from their own commercial ventures ranging from restaurants to industrial health services

**PSI's Top Reseau/100% Jeune/Centre Dushishoze** (Madagascar, Cameroon, Rwanda/1999) *Sexual/Reproductive Health: *Peer counseling; education; contraceptive services; multimedia promotion	Increased contraceptive use among young men from 29% to 53%, among young women from 20 to 39%; increased number of people getting HIV test in Rwanda and reproductive services in Madagascar;	↑ Broad reach through multimedia campaigns and outreach	↑ Provide services at a subsidized rate (Madagascar) and cheaper than other health clinics (Cameroon)	↑ Continuous evaluation to ensure high quality and effective youth programs	Externally- reviewed publications; third-party evaluations	International NGO supported by grants and user fees

**Vision Spring** (India/2001) *Vision correction: *screening, provide glasses, adjustments	"Business in a Bag" strategy allows 1200 Vision Entrepreneurs to distribute > 100,000 pairs of glasses in 13 countries	↑ Entrepreneurs distributed glasses in poor communities and rural areas; door-to-door service with easy screening and testing methods	↑ Glasses are $4 a pair instead of $40-60 at optical shops	↑ Quality of glasses are in general lower than those from expensive optical retailers, but higher than competitors within their price-range	External case studies; externally reviewed publications	Foreign entrepreneurs supported by venture philanthropy, philanthropic investors and user fees

**Ziqitza 1298** (India/2005) *Ambulance Services: *transportation and emergency care; public education	70 ambulances in Mumbai and Kerala have served more than 60,000 patients.	↑ The first single emergency number for ambulance service in Mumbai; 24-hour ambulances with GPS tracking	↑ Cross-subsidization made services more affordable to the poor	↑ 90% of ambulances in urban India did not have adequate equipment and trained paramedics; Ziqitza's ambulances provide trained paramedics, life support equipments and continuous evaluation to ensure safety and quality of services	Self-reported review; funders' review	Local entrepreneurs supported by venture philanthropy and user fees

### Analysis of case studies

Innovations can be characterized within the steps of a health care delivery value chain, as described in Michael Porter and Elizabeth Teisberg's "Redefining Health Care"[13]. A value chain describes each step in a process that adds value to a product or service before it is delivered to the ultimate customer, in this case, a patient. Health care is divided into medical processes (monitoring and preventing disease, diagnosis, intervention, rehabilitation, and ongoing management) and business processes, which support medical care. The value chain served as a starting point for our analysis of the cases, extracting different elements of the business processes, and adding financial functions, which were not included in Porter's original model. We abstracted information on these processes, focusing on those which were described as innovative. We scanned the description of these innovative processes for themes, and after multiple iterations, the categories were restructured to more clearly highlight new strategies used by these organizations to improve care for the poor.

## Results

**Figure 1:**
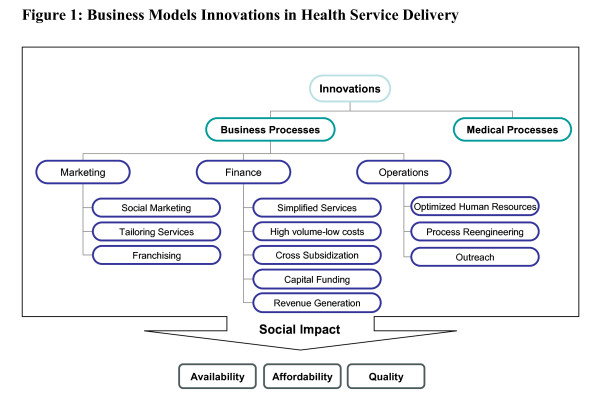
**Business Models Innovations in Health Service Delivery**.

The ten case studies were analyzed using constant comparison of emerging themes, and we found that these organizations had business-process *innovations *in the following functions: marketing, financing, and operating. Figure 1 shows areas of innovation in business processes in the organizations reviewed. Interestingly, all of the organizations innovated across all three categories, with particular strategies described below.

### I. Marketing Activities

The marketing strategies used by many of these organizations included both the promotion of services to the poor and design of these services to meet the needs of this group.

#### Social Marketing

Social marketing refers to the application of marketing techniques to achieve behavioural changes. It is not a new concept, but Population Services International (PSI) in Africa and the Population and Community Development Association (PDA) in Thailand have both applied this strategy in innovative ways. PDA uses Thai humor to address taboo subjects such as contraception and HIV awareness and has achieved unprecedented success in garnering positive public attention[14]. Their social marketing initiatives include "Condom Nights" and "Miss Anti-AIDS Beauty Pageants" in the red light districts of Bangkok. PDA has also established training and peer education programs that focus on behavior change in the country's schools, prisons, sex industry and the public in general. Their condom-distribution network penetrates one-third of Thailand. Their family planning effort contributed to the decrease in the population growth rate in Thailand from 3.3 percent in the 1970 s to 0.6 percent in 2005. The organization developed a national AIDS education program in partnership with government, contributing to Thailand's 90% reduction in new HIV infections in 2004. PSI, meanwhile, operates three social marketing programs that offer educational programs on reproductive health for urban youth in Africa. The programs address the taboo subject of safe sexual behavior through means that target the youth, such as magazines, television spots, call-in radio shows and radio drama[13]. A survey found that 90% of the youth had read the monthly magazine at least once and 70% had viewed the television spots, with corresponding increased rates of contraceptive use and HIV testing, demonstrating the potential of these educational social marketing programs [15].

#### Tailoring services to the poor

Another marketing tool employed was tailoring the design of products and services to the needs of the poor. The Bhagwan Mahaveer Viklang Sahayata Samiti (BMVSS) is an Indian organization that has developed the Jaipur Foot, an artificial lower limb prosthetic intended to meet the needs of amputees living in developing nations, where squatting, sitting cross-legged and walking barefoot is common to the poor but largely impossible with typical prosthetic limbs[16]. In addition to providing a novel product, the BMVSS clinics have adapted their services to the poor, allowing patients to check-in at any time of day or night. Furthermore, they provide patients with free room and board if they have to spend the night and provide their families with free meals at the clinic. Since fittings can be completed in one session (as opposed to several), time away from work and number of visits are kept to a minimum, which is very important to patients with limited means and mobility. This specific tailoring of products and services make them more accessible and attractive to the poor.

#### Franchising

Franchising has been used to facilitate rapid expansion and the sustainable distribution of products and services of a specified quality in reproductive health. Greenstar Social Marketing Pakistan is one of the first health franchisers, and has grown to provide over 26% of all contraceptives in Pakistan. It targets low-income non-users of contraceptives through a total market approach, which has different price points for each segment of the population[17]. The organization operates a franchise network of over 7,500 private independent health care providers, most of which are located in low-income urban and peri-urban areas in Pakistan. Greenstar signs franchising agreements with providers for distribution of products or social services, and keeps regular contact with the aim of ensuring adequate quality. It provides medical training, supply of goods, public education, technical support, quality control and program evaluation to its franchisees. Greenstar has invested in developing a strong brand associated with high quality care and reliable information.

### II. Financial Strategies

Most organizations in this study were funded by local entrepreneurs who wanted to make an impact on society, while two of the organizations initially received funds from international NGOs and have later grown to be more independent (see Table 1). Many received support from partnerships, government funding, grants and donations, and some recovered part of their costs from user fees. Our analysis of these cases revealed that while some organizations innovated to generate funds for sustainability, many organizations redesigned cost structures in ways that allowed products and services to be more affordable to the poor. Dramatic reductions in cost were reported to have been achieved by rigorous expense management, capital funding, and revenue-generating programs.

#### Lower operating costs through simplified medical services

Operating costs were lowered by simplifying the medical services provided and using less than fully qualified providers. For example, VisionSpring's financial strategies include a "business in a bag", which involves training rural community members to become Vision Entrepreneurs (VEs) who can provide vision screening, identify far-sightedness and provide glasses for vision correction. VEs are provided a kit with items intended to help launch a business, including multiple styles, colors, and powers of reading glasses, screening equipment and marketing materials[17]. VisionSpring helps replenish supplies of reading glasses and provides additional support as required. This "business in a bag" strategy is intended to enable motivated workers to gain access to an entrepreneurial opportunity without the barriers of high set-up and operating costs.

#### High volume and low unit costs

The Narayana Hrudayalaya Heart Hospital (NH) in India, the largest provider of pediatric heart surgeries in the world, has reduced the unit cost of cardiac surgeries through volume (they do eight times more surgeries per day than the Indian average), which maximizes the use of infrastructure [18,19]. The Hospital rents machines for blood tests and pays only for reagents, which satisfies suppliers given the high volumes. NH reduces cost by relying on digital X-rays rather than expensive films and by reducing inventory and processing times using comprehensive hospital management software. The quality of care has not been compromised by the high volume. In fact, NH uses high volume to improve the quality of care by allowing individual doctors to specialize in one or two types of cardiac surgeries. Their success rates are high (1.4% mortality rate within 30 days of coronary artery bypass graft surgery v.s. 1.9% in the U.S)[20]. NH's average cost of open heart surgery is about $2,000 USD, for which NH charges $2,400 as compared to $5,500 in the average Indian private hospital [21]. A third of the patients actually do not pay out of pocket. The founder of NH partnered with the state of Karnataka to start the farmer's insurance plan, which costs $3 a year per person and reimburses the hospital $1200 for each surgery. The hospital makes up the difference by charging more from the 40% of patients who do not have a plan and the 30% who opt for private/semi-private rooms [20]. NH's high volume and tiered-fee strategies allow them to provide affordable quality heart surgery to the poor. The next section will address tiered-fee strategies in more detail.

#### Cross-subsidization

Some organizations have achieved financial sustainability through a cross-subsidization strategy, where they exploit the greater willingness and ability to pay amongst the wealthier patients to cross-subsidize expensive services for lower-income patients. They have developed efficient ways of assessing financial need and implementing cross-subsidy. Aravind Eye Care System, the largest eye care provider in the world, attracts wealthier patients who pay market rates and then provides the same services for the poorer 70% of their patients at a highly subsidized rate or for free [21-23]. They establish differential pricing by the patients' choice of amenities and the type of lens to be inserted in the eye, not by the quality of treatment the patient gets. All patients - regardless of ability to pay - receive the same medical care, but paying patients can choose soft lenses and sleep in private rooms, while non-paying patients are given the basic hard lens and sleep in open dormitories on mats. This approach, called quality targeting, is an efficient way of assessing financial need because those who can afford private rooms and soft lenses are much more likely to choose them. Another example is 1298 Ziqitza Health Care Limited, which provides private ambulance services using a tiered fee system [24]. Patients call the ambulance service and are charged according to the hospital they have arranged to be transported to - those going to private hospitals are charged above cost, those going to free government hospitals pay a nominal fee, and trauma patients do not pay. In this strategy, a patient's ability to pay is gauged from the choice of hospital, and again patient's have an incentive to accurately represent their ability to pay because it impacts the quality of hospital care they receive subsequently. Approximately 20% of patients carried by the ambulance service over the last three years were subsidized, allowing Ziqitza to be financially sustainable.

In addition to formal tiered payment systems described above, an informal system of cross-subsidy can be created by encouraging providers to provide subsidized services to poor people. Dentista Do Bem is a large network of private, for-profit dentists in Brazil who have agreed to see a few poor patients every day for free [25]. This is a form of charity that has a limited impact on the earnings of for-profit providers, with paying customers indirectly "subsidizing" the cost of caring for poor patients within a given practice. Children are screened in schools and recruited to join the program until age 18. Though each dentist only sees a few free patients a day, the large number of participating dentists made it possible to see more than 12,000 children in 2009 [7,25]. Providers derive some satisfaction and recognition for providing this service and the network is an efficient organizational structure to leverage existing human resources to reach poor people across all 27 Brazilian states, and in 6 Latin American countries [26].

#### Capital Funding

Capital funding for franchisees or service providers to start up or improve the quality of their health programs is a key feature of the Kisumu Medical and Educational Trust (KMET), a franchise which gives training in reproductive health to private providers in Kenya[27]. KMET improves the availability of funds to private franchisees through revolving loan programs (microfinance). This allows community-based providers to expand services and improve the quality of the reproductive health services offered. KMET has expanded to 125 franchisees since startup in 1995.

#### Generating Revenue

Thailand's PDA developed 16 for-profit companies that are affiliated with the organization and are mandated to put funds towards the NGO to facilitate expansion and supplement operating costs. One of PDA's many innovative commercial ventures is the "Cabbages and Condoms" Restaurants, located in different parts of the country, where condom-themed food and drink help bring money into the organization[28]. This unique set-up allows the companies to independently generate revenue while using novel social franchising mechanisms to spread information about safe-sex practices

### III. Operating Activities

These health care organizations appear able to modify operating strategies to increase the availability of services in remote areas and make judicious use of human resources in a context of widespread shortage of skilled labour.

#### Optimizing human resources

While this strategy is not unique, these organizations have expanded the use of lay health workers into new areas. They help laypeople acquire skills that were previously exclusive to trained professionals: distribution of oral contraceptives (PDA) or eye exams and business operations (VisionSpring). By shifting tasks to trained lay people, these organizations have reduced operating costs, increased availability of staff, and empowered the local community. Aravind Eye Care System trains high school graduates from rural areas into paramedical staff like patient flow managers, providers of simple diagnostic procedures, and even optical technicians[21]. Another approach for leveraging human resources is increasing the quality of care provided by established health care workers. KMET trains existing health workers in safe abortion procedures and provides manual vacuum aspiration kits for safe abortions. Education, resources, and a professional network are designed to further enhance the quality of maternal and child care given by this group[27].

#### Process and product reengineering

In addition to distributing ready-made eye-glasses for the far-sighted, VisionSpring is working together with the d.o.b foundation to offer new adjustable lens (U-specs) for the near-sighted population, and especially for children[29]. The innovative design of U-specs comprises of two adjustable lenses that can be shifted to adjust the refractive strength of glasses. This makes mass production easier, reduces costs and offers an alternative to the traditional customized construction of eye-glasses.

Aravind Eye Care System improved efficiency by reengineering their operating rooms to allow surgeons to work on two tables in alternation by shifting from one case to another. While one surgery is in progress, a team of 4 nurses and paramedical staff prepare the next patient. This innovation allows Aravind to perform a cataract surgery in 10 minutes - one third of the industry standard of 30 minutes. Despite the shared space for patients, their infection rates are 4 per 10,000 cases, which is better than the published rate in the UK of 6 per 10,000 [23]. Aravind also tracks surgical outcomes by surgeon and provides support to those who are below average, which contributes to improvements in quality of care.

#### Increasing outreach

Aravind Eye Care System and Narayana Hrudayalaya Heart Hospital provide health camps to reach patients in rural areas. NH provides camps that focus on cardiac diagnosis with transportation to the hospital for patients who require it. In addition to health camps, Aravind has also set up internet kiosks in remote villages run by community members, who take pictures of patients' eyes using a webcam and send the images to a doctor from Aravind along with a completed online questionnaire about the patients' symptoms [7]. The doctor is able to access the images instantaneously, and chat with the patient online in real time to assess whether the patient requires consultation at the hospital. These kiosks reduce both the time and expense incurred by an unnecessary hospital visit.

## Discussion

This study characterized 10 high-profile private sector innovators which have improved health services for the poor, reviewed their strategies and found several trends across the organizations.

### Complete marketing, finance, and operations solutions

Analysis of each organization's strategy showed that they innovated across marketing, finance, and operations. Exemplary practices include patient-experience-focused strategies such as tailoring designs and services to meet the needs of the poor and cross-subsidization, efficiency strategies such as specialization and high-volume/low cost approaches, and operational approaches to increase availability of services, such as outreach and telemedicine. All of the organizations that we studied had at least one unique innovation in each of the key business processes. There appears to be no single effective approach to improve health delivery. This may serve as a caution to organizations looking for "silver bullets" to improve care for the poor. The World Bank's recent review also showed that there are no blueprint planning approaches for improving the performance of health organizations [30]. In fact, each exemplar in our study has developed a novel and comprehensive approach, simultaneously addressing the fact that poor people are often unaware of services, have limited funds and live in hard-to-reach areas. This finding is similar to the Karamchand et al.'s stating that social service organizations that have scaled up successfully in emerging markets provide "end to end solutions"[9]

### Narrow clinical focus

All of the organizations in our study had a narrow disease focus built around a few medical processes with multiple innovations allowing them to market their services on a large scale, reduce costs, and streamline operations to target poor patients effectively. While this may be an artifact of our search strategy, we found that none of the organizations identified here provided broad-based comprehensive health services. This finding could be related to the fact that it is easier to manage and experiment within well-defined health care delivery systems with a narrow focus. The predictability of the health problems and treatment strategies make it easier to simplify processes, delegate tasks to lower trained personnel and measure quality, all of which can reduce costs while increasing reach and quality. Though vertical approaches have limitations, they may lead to innovations whose benefits could be captured by replication or by linking them to broad-based health services, as in the case of PDA's collaboration with the Thai government on HIV control. The partial integration of PDA's program into health system functions contributed to a nation-wide reduction of the HIV infection rate. In fact, studies show that seldom are interventions wholly unintegrated (purely vertical) or fully integrated into health system functions, and the heterogeneity in the extent of integration is influenced by intervention complexity, health system characteristics and contextual factors [31]. Since the organizations chosen for our study vary by disease area, geographical, economic and political environment, there is no doubt that the intent and extent of integration of these targeted health interventions into the health system, if any, will also vary.

### Disruptive Innovations

Some of the organizations we studied have designed "disruptive" services and products designed to enable the participation of poor consumers who were previously excluded. For example, Jaipur Foot developed an artificial foot that is affordable, easy to fit and has functions better suited to the needs of the poor. VisionSpring provides readymade reading glasses on the spot to customers, using a kit that has very simple eye-screening equipments and procedures, such as threading a needle. These customer-oriented products and services are "disruptive" in the sense that they fill gaps in the conventional markets, but have not yet displaced previous approaches. Both Jaipur Foor and VisionSpring have effectively increased accessibility to medical services to the poor through their simple and affordable designs. The vertical approaches described above could also be described as incorporating a value-added process business model, which allows for the refinements in quality while reducing cost through simplification and delegation of certain processes to less skilled providers [32]. For the most part, they do not pursue a low cost, low quality service strategy. Most organizations adapt services to the needs of their clients, and some reduce the "frills" but aim to provide high quality clinical care.

### Business process innovation

The core innovations of most organizations we reviewed are in business rather than medical processes, demonstrating that it is possible to have large scale impact by implementing existing care processes using innovative marketing, finance and operating strategies. For example, PDA's national success relies strongly on its innovative marketing campaigns for family planning and HIV prevention in Thailand. Ziqitza's ambulance services use standard protocols, but achieve financial sustainability and affordability through its novel approach to cross-subsidy. Given the wide range of affordable and effective medical interventions which are currently underused,[33] it seems that many of the problems in global health require improvements in management rather than new interventions. Some of the strategies described here have been successfully reproduced, by the Thai government in the case of PDA, and by other hospitals using the consulting services of Aravind Eye Care System [21]. This suggests that the private sector may be a viable source of innovative management practices.

One of the main concerns with heath care delivery from exemplars of private sector innovation is the issue of quality. In our study, quality of care was rarely compared to existing services, and improvements in quality were only measured for a few organizations. Some organizations focus on affordability like Dentista Do Bem, which presumably did not improve availability or quality of care since they leverage existing providers. PDA did not provide any evidence on quality of their family planning or HIV educational programs, but the national scale up of their strategy coincided with a significant decrease in population growth rate in Thailand, which indirectly suggests some social impact. The only organizations who had more rigorous evaluations of quality of services were Aravind Eye Care System, PSI, Greenstar and NH. The quality of care for the rest was inferred through changes in structure, like built-in quality improvement mechanisms, training, monitoring and evaluation. Despite having chosen among the best documented organizations, there is a lack of rigorous evidence for many measures of impact. Future work should focus on improving data collection for impact assessment, encouraging third-party appraisal, and possibly reinforcing evaluations by changing funding requirements where relevant.

Due to the nature of our search strategy, we are only able to capture organizations that are relatively well-documented and high-profile, and some worthy innovators who are less successful at marketing their story might have fallen under the radar. Another limitation to this study is establishing what is truly innovative. For this study, we relied on reputation and a review of organization for which data was available rather than a systematic review of all existing organizations to ensure that there was no overlap. However, high-profile innovative organizations that have scaled up their operations are likely to be copied, in which case they may not be the only ones using a given strategy at this time. For example, Aravind Eye Care System contributed to the development of the Lumbini Eye Institute, which operates on a similar model and now provides 25% of all sight restoring surgeries in Nepal. More in-depth studies are required to assess associations between a given strategy and social impact. The organizations identified here should not be seen as representative of the private sector in general, rather they were selected as exemplars of what this sector might contribute. They may in fact be islands of excellence in a sea of mediocrity, though it was beyond the scope of this study to determine if this was the case.

This is the first study to characterize and compare a wide range of activities among the best documented health care organizations into a coherent framework. Unlike previous studies, this study focused on health care, included all countries in the initial search (as opposed to only India or Africa), and looked for patterns across a series of cases sampled for maximum variability. This study is not an attempt to build a complete database of innovative private sector providers (like the Center for Health Market Innovation), but rather an attempt to lay the groundwork for larger studies to determine the association between a given strategy and improved outcomes. With increasing investment in social enterprises from groups like Acumen Fund and the Global Impact Investing Network, more attention to rigorously measure the impact of these organizations would be beneficial. An independent group like the International Initiative for Impact Evaluation could develop appropriate metrics and provide a platform to independently and reliably assess the impact of organizations who receive funds from impact investors or government, especially around quality of care. Researchers could work with these investors to evaluate social impact and develop reliable measures that are appropriate to organizations that are scaling up quickly, since many evaluation designs provide results too slowly to assess effective growth.

## Conclusion

The poor in low and middle income countries have limited access to quality health services for a variety of reasons. A subset of private health organizations have emerged, often called social enterprises, which have developed innovative techniques to improve care for the poor. In this review, ten high-profile health service organizations were studied, and were found to innovate across the areas of marketing, finance, and operation. Rather than providing a wide range of services, these organizations had a narrow clinical focus, which may have facilitated experimentation with delivery processes. This review of many of the best-known innovators in health services for the poor found relatively little rigorous information on quality of care. Linking future investment to robust measures of social impact would help identify effective approaches and unleash the potential of innovative delivery models to transform health services for the poor.

## Authors' contributions

All of the authors contributed to the design of the study, OB and SK carried out the primary literature review and surveys, and initial analysis, with feedback from AM, DD, AD, and PS. All of the authors reviewed and commented on the initial results. OB wrote the first draft, with substantial comments and revisions by the other authors. They also commented on subsequent drafts. All authors read and approved the final manuscript.

## Conflict of interest

The authors declare that they have no competing interests.

## Supplementary Material

Additional file 1 **Search Strategy**. List of search terms used and databases consulted in the review process. Click here for file
