# Supplementary material for: Innovative health service delivery models in low and middle income countries - what can we learn from the private sector?
Source: Health Res Policy Syst. 2010 Jul 15;8:24. doi: 10.1186/1478-4505-8-24 (PMC3236300; doi:10.1186/1478-4505-8-24)
Supplement: Additional file 1 — Search Strategy. List of search terms used and databases consulted in the review process. [file 1478-4505-8-24-S1-1.docx]

**Appendix 1: Search Strategy**

***Types of literature searched***

• Peer-reviewed journal articles

• Gray literature

• Project reports and documents on Web sites of organizations

• News articles

• Documents provided by expert sources

***Databases consulted***

• Medline

• Academic Search premier

• PubMed

• EMBASE

• Web of Science

• HealthSTAR

• POPLINE

***Web sites and sources consulted***

• U.S. Agency for International Development (www.usaid.org)

• World Health Organization (www.who.int)

• Population Services International (www.psi.org)

• President’s Emergency Plan for AIDS Relief (www.pepfar.gov)

• Ashoka (www.ashoka.org)

• The Skoll Foundation (www.skollfoundation.org)

• The Schwab Foundation for Social Entrepreneurship (www.schwabfound.org)

• The Acumen Fund (www.acumenfund.org)

• London School of Hygiene and Tropical Medicine (www.lshtm.ac.uk)

• BBC News (http://news.bbc.co.uk)

• International Finance Corporation Report (IFC 2008)

• Local news Web sites

• Organizational Web sites (where available)

• Conversations with experts in the field

***Search limits***

• Journal articles were limited to articles in English, but organizational Web sites

were limited to English, French, and Chinese.

• Geography limitations were to low- and middle-income countries.

• Program limitations included purely public sector programs and programs that fell

outside the realm of health delivery.

***Search terms included but were not limited to***

• Health service delivery developing country

• Innovative health service delivery

• Health franchising

• Social marketing

• Health training

• Human Resources for health

• Human Resource Retention Health

• Innovative Health Africa

• Innovative Health India

• Innovative Health Asia

• Public Private Partnerships Health

• Private Sector Health Service Delivery

• Health and technology

• Community based care

• Health delivery increased quality

• Health delivery increased availability

• Health delivery increased affordability

• Employer health service delivery

• High volume low cost hospitals

• Health integrated networks
